# Predicting respiration rate in unrestrained dairy cows using image analysis and fast Fourier transform

**DOI:** 10.3168/jdsc.2023-0442

**Published:** 2023-11-17

**Authors:** Raphael R. Mantovani, Guilherme L. Menezes, João R.R. Dórea

**Affiliations:** 1 Department of Animal and Dairy Sciences, University of Wisconsin–Madison, Madison, WI 53706; 2 Department of Biological Systems Engineering, University of Wisconsin–Madison, Madison, WI 53706

## Abstract

Respiratory rate (RR) is commonly employed for identifying animals experiencing heat-stress conditions and respiratory diseases. Recent advancements in computer vision algorithms have enabled the estimation of the RR in dairy cows through image-based approaches, with a primary focus on standing positions, thermal imaging, and deep learning techniques. In this study, our objective was to develop a system capable of accurately predicting the RR of lying Holstein cows under unrestrained conditions using red, green, and blue (RGB) and infrared (IR) night vision images. Thirty lactating cows were continuously recorded for 12 h per day over a 3-d period, capturing at least one 30-s video segment of each cow during lying time. A total of 95 videos were manually annotated with rectangular bounding boxes encompassing the flank area (region of interest; ROI) of the lying cows. For future applications, we trained a model for ROI identification using YOLOv8 to avoid manual annotations. The observed RR was determined by visual counting of breaths in each video. To predict the RR, we devised an image processing pipeline involving (1) capturing the ROI for the entire video, (2) reshaping the pixel intensity of each image channel into a 2-dimensional object and calculating its per-frame mean, (3) applying fast Fourier transform (FFT) to the average pixel intensity vector, (4) filtering frequencies specifically associated with respiratory movements, and (5) executing inverse FFT on the denoized data and identifying peaks on the resulting plot, with the count of peaks serving as the predicted RR per minute. The evaluation metrics, root mean squared error of prediction (RMSEP) and R^2^, yielded values of 8.3 breaths/min (17.1% of the mean RR) and 0.77, respectively. To further validate the method, an additional dataset comprising preweaning dairy calves was used, consisting of 42 observations from 25 calves. The RMSEP and R^2^ values for this dataset were 13.0 breaths/min and 0.73, respectively. The model trained to identify the ROI exhibited a precision of 100%, a recall of 71.8%, and an *F*_1_ score of 83.6% for bounding box detection. These are promising results for the implementation of this pipeline in future studies. The application of FFT to signals acquired from both RGB and IR images proved to be an effective and accurate method for computing the RR of cows in unrestrained conditions.

## Highlights

• Fast Fourier transform was used to predict respiration rate.
• Computer vision was used to evaluate respiration rate in cows and calves.
• Respiratory rate prediction may help in diagnosing heat stress and respiratory disease.
• The model is capable of predicting respiration rate without training.

## Graphical Abstract

**Figure:**
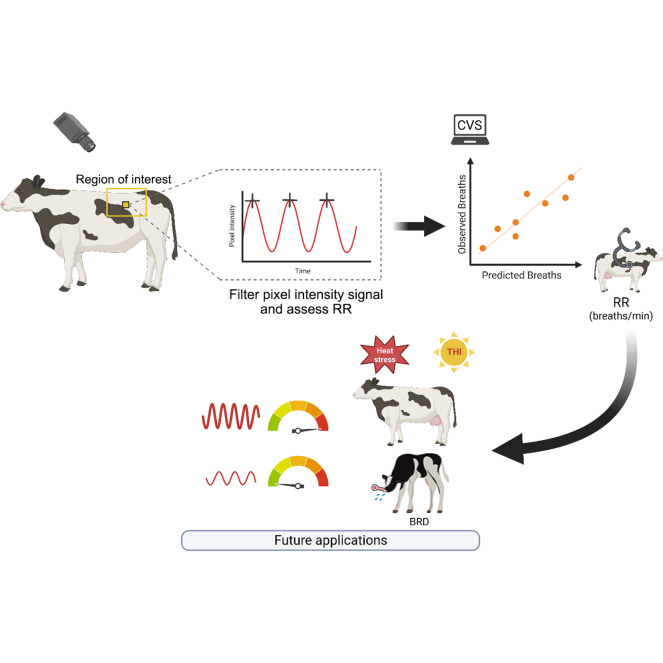
**Summary:** Respiratory rate (RR) plays a crucial role in monitoring the health of livestock and serves as an indicator for assessing heat stress and respiratory diseases. However, the traditional approach to calculating the RR in cattle involves visually observing movements in the flank area, which is a labor-intensive method with limited scalability. In this study, we have developed an automated technique that uses image analysis and fast Fourier transform to capture the RR of unrestrained, lying dairy cows. Our results demonstrate that this proposed technology offers a faster and more scalable alternative for monitoring RR in cattle.

Respiratory rate (**RR**) is an important trait associated with animal physiology and has been commonly used to assess the health status of livestock (Jorquera-Chavez et al., 2019) and humans (Kakouche et al., 2021). Multiple studies have used this feature as an indicator of heat stress, respiratory diseases, and animal welfare for dairy cattle (Gaughan et al., 2000; Li et al., 2020), pigs (Jorquera-Chavez et al., 2021), and horses (Rammerstorfer et al., 2001). This has made calculating the RR a central concern for precision livestock, which is why many methods have been developed to calculate it.

Traditionally, the RR has been evaluated through the visual inspection of movements in a cow's flank area (Shu et al., 2021). This method, however, is not only labor intensive but also requires specific training and does not scale well, thereby limiting its use in large-scale operations (Handa and Peschel, 2022). To overcome these limitations, several studies have recommended the application of automated technologies such as wearable sensors (Eigenberg et al., 2002), infrared thermal imaging from the nostrils (Stewart et al., 2017; Lowe et al., 2019), and red, green, and blue (**RGB**) imaging of abdominal movements (Wu et al., 2020, 2023) to assess RR in livestock. Since wearable sensor technologies are typically invasive and do not enable real-time RR monitoring (Wu et al., 2020), video-based techniques have presented various benefits, such as lower costs, enhanced scalability, reduced risk of physical damage, and minimized stress on the animals (Handa and Peschel, 2022).

Infrared thermal cameras have demonstrated efficacy in capturing the patterns of air inhaled and exhaled through an animal's nostrils. This capability suggests promising results for calculating RR in research applications (Stewart et al., 2017; Lowe et al., 2019). However, implementing this technology in commercial settings presents several challenges. Specifically, these cameras need to be positioned close to the object of interest, unless high resolution cameras are deployed, which can be costly. Additionally, the environmental temperature can affect the camera measurements, resulting in noisy images (Gade and Moeslund, 2014), and thermal imaging cameras are generally more expensive than simple RGB (surveillance) cameras. For research studies focusing on the animal's head as the region of interest, precise positioning of the head is required for RR calculation, making it a challenging task due to the animal's natural movements during handling (Shojaeipour et al., 2021).

In this context, some researchers have used cameras to record the flank area, a region commonly observed during visual inspection for calculating RR. Wu et al. (2020, 2023) showed promising results for tracking RR through RGB videos in dairy cows. Nevertheless, deep learning methods typically need large datasets collected across diverse environments to achieve robust generalization. Therefore, using fast Fourier transform (**FFT**), a computationally efficient algorithm that breaks down signals into their constituent frequencies, to process the respiratory movements captured via video could be an efficient analytical alternative for monitoring RR in animals. For instance, Wiede et al. (2017) demonstrated that the use of FFT to analyze the average pixel intensity variation over the abdominal area in breathing humans can effectively calculate their RR. However, the environmental conditions for tracking flank area movements in cattle are often more uncontrolled and unpredictable than in humans. This raises the question of whether the same algorithm could accurately predict the RR in cattle, given the potentially noisy signal obtained from unrestrained animals.

In light of the above-mentioned technologies and their limitations, this study aimed to apply FFT to the average pixel intensity of both RGB and infrared (**IR**) videos featuring unrestrained, lying dairy cows to assess their RR. The objective was to devise a straightforward yet robust model capable of predicting RR via image analysis. The proposed approach has potential value for automatic RR detection in large-scale dairy farming, contributing to the early identification of cows experiencing heat stress, exhibiting abnormal respiratory behaviors, or both.

The videos for this study were collected in July 2021 at the Dairy Cattle Center of the University of Wisconsin–Madison. Approval for all animal evaluations was granted by the Institutional Animal Care and Use Committee of the University of Wisconsin–Madison, under protocol number A006380. The facility had a combination of artificial and natural lighting throughout all RGB recordings, whereas the night vision videos (IR recordings) had no light. Videos in both RGB and IR were recorded using an Amcrest ASH42-W camera (Amcrest Technologies), which was set at a frame rate of 30 frames per second and had a resolution of 2,560 × 1,440 pixels. These cameras include infrared light-emitting diodes, enabling night vision up to 30 m. The cameras were positioned approximately 2 m above the ground and 5 m away from the cows' flanks, capturing 1 to 4 resting Holstein cows at an angle of approximately 65°. Recordings spanned 12 h (1800 to 0600 h) over 3 d, resulting in at least one 30-s video segment of each cow obtained daily. In total, 95 videos and 193 observations of the subjects were recorded. The ground truth data (observed data) were collected by 2 observers who visually counted the RR and converted it to breaths per minute.

After the videos were collected, one frame was extracted from each recording. These images were then exported to the VGG Image Annotator (Dutta and Zisserman, 2019), where a rectangular bounding box was annotated over the cows' flank areas. Said annotations represented the region of interest (**ROI**) where respiration could be observed. Subsequently, the videos and their corresponding annotated ROI were imported into Python using the Open cv2 library, and the average pixel intensity for each frame and channel (R, G, B) was calculated. A summary of the processing pipeline can be found in Figure 1.

**Figure 1:**
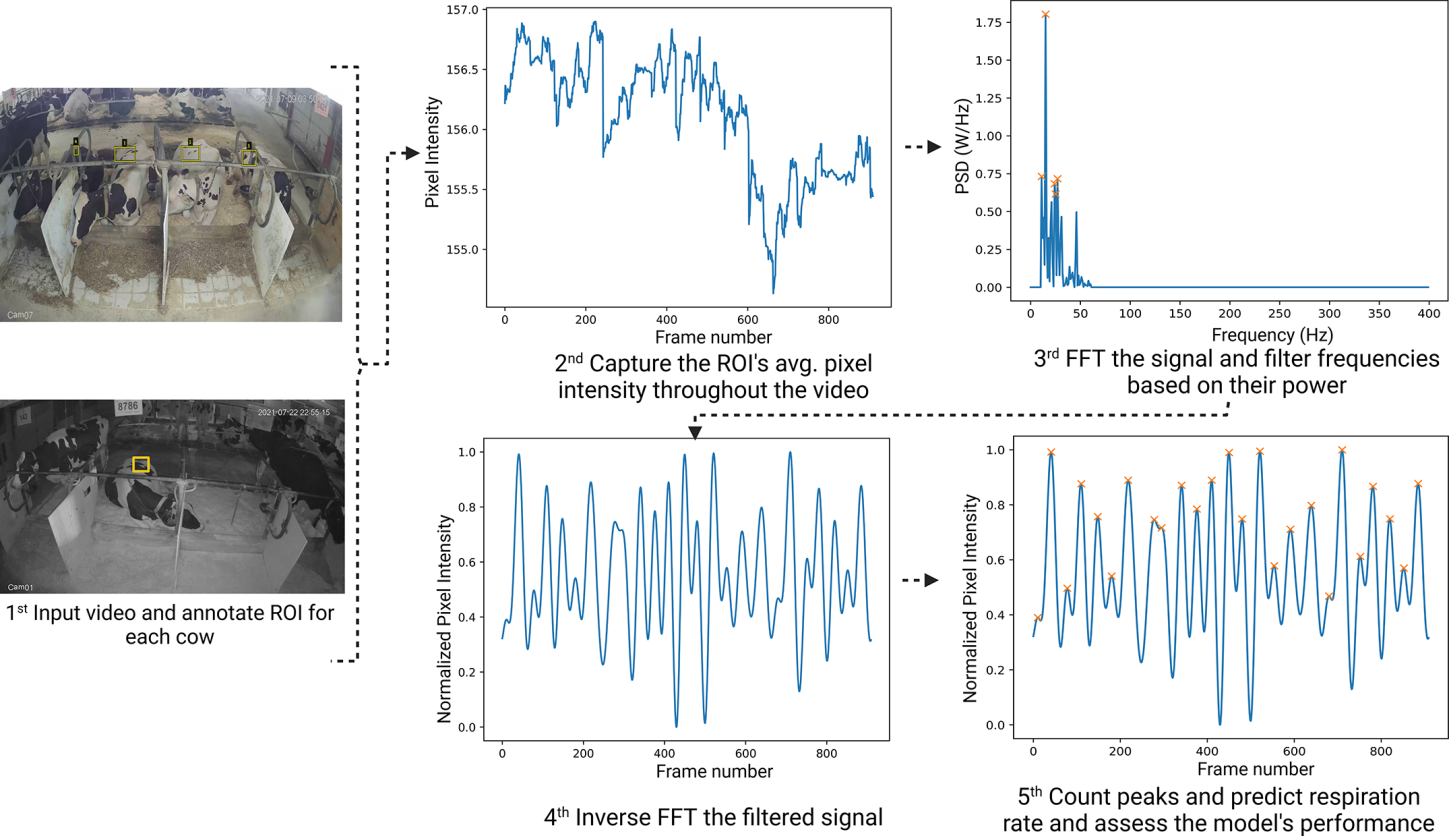
Summary of the proposed method. First image: examples of a red, green, and blue (RGB) and an infrared image captured for the cows with the annotations made. Second image: average pixel intensity variation over the annotated region of interest (ROI). Third image: power spectral density (PSD) of each frequency in the data after performing fast Fourier transformation (FFT); the 5 selected frequencies are represented with an orange ×. Fourth and fifth images: denoized signal after filtering and the peak count performed to calculate the respiration rate.

The frequency domain of the 2-dimensional pixel-intensity signal was obtained using the FFT. To compute the final RR, 2 preprocessing steps were implemented to eliminate signal noise. First, only frequencies (Hz) between one-third and twice the video length were selected, since the RR of healthy and moderately heat-stressed cows (which represented the conditions of this study) typically range from 26 to 120 breaths per minute (Becker et al., 2021). Second, we selected only the frequencies with the top n power spectral densities (**PSD**).

To determine the top n PSD values for selecting respiration-related frequencies, the dataset was divided into 3 separate training and testing groups. In each division, the training set encompassed all observations from 20 cows, whereas the testing set comprised all observations from the remaining 10 cows. The top n PSD values were considered hyperparameters in the model, and tests were conducted for n = 50, 25, 13, 5, 3, 1. For instance, n = 50 indicates the top 50 values of the PSD. The values were evaluated for each training and testing set, and the root mean squared error of prediction (**RMSEP**) and R^2^ were calculated accordingly. Subsequently, the n value that resulted in the best performance (lower RMSEP and higher R^2^) was used to perform the data filtering on the entire dataset.

After performing these processing methods, the cleaned data were transformed back to their original unit (pixel intensity) using the inverse FFT and normalized to a scale between 0 and 1 using min-max normalization. Finally, the peaks in the resulting signal were identified using the SciPy library, and the peak count was used as the predicted respiration rate, expressed in breaths per minute. The function used to assess respiration rate was scipy.signal.find_peaks, keeping all its default parameters. Hence, the input (x) was the inverse-Fourier-Transformed data, the rel_height was equal to 0.5, and the remaining parameters (height, threshold, distance, prominence, width, wlen, and plateau_size) were left as “None.”

After collecting the predicted RR values, the best combination of PSD was selected to predict RR, and the Z-scores were calculated to identify any significantly (*P* < 0.01) outlying results within the data. It was observed that one video exhibited a Z-score greater than 5 (*P* < 0.001), indicating a possible outlier. Upon further examination of the video, it was determined that the presence of a bright light beam flashing over the ROI where the cows were lying caused this anomalous error. As a result, this video was excluded from the model's analyses. Consequently, the dataset used to assess the model's performance consisted of 94 videos and 191 observations. The metrics used to evaluate the model's performance were the R^2^ and the RMSEP.

Given that the proposed method relies on variations in pixel intensity throughout the videos, it was deemed crucial to consider the potential impact of lighting changes and non-respiration-related movements on its robustness. To address this concern, an additional analysis was conducted using a subset of the original dataset, which consisted of 170 observations. A total of 21 videos were excluded due to various issues: cow movements compromised 11 videos (leg motions in 7, hook shifts in 2, head movements in 2); lighting issues affected 6 (lack of flank visibility in night recordings in 5, insect interference in 1); and ROI occlusion led to the exclusion of 4 videos (tube in the tiestall obstruction in 3, adjacent cow in 1). These exclusions were pivotal as they introduced noise and altered the ROI, thereby corrupting the depiction of respiratory movements. Meanwhile, videos with less significant movements (e.g., head or leg movements without a change in the ROI's position) were retained, as they did not displace the ROI from the flank area. Two human observers were employed to ensure the videos met the filtering criteria. The performance metrics analyzed were the same as those in the previous analysis.

Finally, the proposed model was also evaluated on an additional dataset comprising 42 videos from 25 calves, which was used to assess the performance of the model on these animals. The videos were recorded at a commercial dairy farm in Wisconsin. The camera specifications and data collection protocols aligned with those described in the preceding sections. The video segments were captured from an almost top-down perspective. The evaluation metrics employed for analysis were R^2^ and RMSEP, which were consistent with those used for the Holstein cows.

Having analyzed the training and testing sets to determine the optimal number of top n PSD values for accurately capturing the RR, it was found that selecting the top 5 values consistently resulted in the best predictions across all testing sets. These findings are presented in Table 1, which clearly demonstrates that the top 5 PSD values achieved the highest R^2^ values and the lowest RMSEP values for each dataset. As a result, the data were filtered, and only the 5 most prominent frequencies were selected to compose the cleaned signal, as depicted in the third image in Figure 1.

**Table 1:** Model performance metrics for every top n power spectral density value tested in each different testing and training set

Dataset^2^	N	Power spectral density^1^											
		Top 50		Top 25		Top 13		Top 5		Top 3		Top 1	
		R^2^	RMSEP	R^2^	RMSEP	R^2^	RMSEP	R^2^	RMSEP	R^2^	RMSEP	R^2^	RMSEP
Train 1	59	0.36	21.1	0.39	18.8	0.65	12.1	0.78	8.4	0.60	10.7	0.32	15.7
Test 1	36	0.33	19.2	0.31	18.3	0.65	11.1	0.76	8.0	0.61	9.6	0.39	12.6
Train 2	65	0.37	20.3	0.40	18.2	0.68	11.5	0.78	8.1	0.56	11.0	0.30	15.3
Test 2	30	0.31	21.0	0.32	19.3	0.61	12.2	0.75	8.5	0.68	9.2	0.41	13.8
Train 3	66	0.31	20.2	0.31	18.9	0.62	11.7	0.75	8.2	0.64	9.4	0.40	13.2
Test 3	29	0.41	21.3	0.48	18.1	0.70	12.0	0.80	8.3	0.53	12.3	0.23	17.8

1 n = total number of observations (videos); RMSEP = root squares mean prediction error (breaths/min).

2 Training and test 1 were defined with 20 and 10 cows, respectively.

The original and cleaned signals for an arbitrary animal are presented in the second and fourth image of Figure 1. In the majority of cases, the method effectively identified the constituent frequencies of the respiratory movements, resulting in a denoized signal that facilitated the estimation of the RR. Furthermore, as shown in the fifth image described in Figure 1, the application of peak counting on the cleaned signal using the Python function scipy.signal.find_peaks accurately captured the respiratory signals of the animals. This finding is consistent with the research conducted by Anishchenko et al. (2019), wherein the same function was used to estimate the RR of humans using bioradar signals and yielded satisfying results.

For future applications, we trained ROI identification using YOLOv8 (Glenn, 2023). A total of 1,150 images were extracted from 95 different videos, which were captured by 10 different cameras and manually annotated. Seven cameras were selected for the training set, providing 850 images, whereas 3 different cameras capturing different cows were used for the testing set, which consisted of 300 images. The input image size was set to 640 × 640, the batch size to 32, and the network was trained for 200 epochs. The ROI model was implemented on a Linux server with 40 GB of RAM and an NVIDIA A100 GPU.

The prediction results for the 30 dairy cows are depicted in Figure 2. The method demonstrated an overall performance with an R^2^ value of 0.77, indicating the correlation between the predicted and observed number of breaths in the cows over a 30-s video segment. The RMSEP was 8.3 breaths per minute, equivalent to 17.1% of the mean predicted respiration rate. When assessing precision in RGB and IR videos, the model exhibited slightly superior performance in RGB videos (R^2^ = 0.81) compared with IR (R^2^ = 0.74). It is noteworthy that the dataset consisted of 79 RGB videos and 112 IR videos. These findings suggest that the night vision conditions posed more demanding lighting conditions for capturing the RR. Nevertheless, the method consistently generated accurate predictions, aligning with the findings of Wiede et al. (2017), who demonstrated the precise capture of RR through pixel intensity monitoring in RGB images.

**Figure 2:**
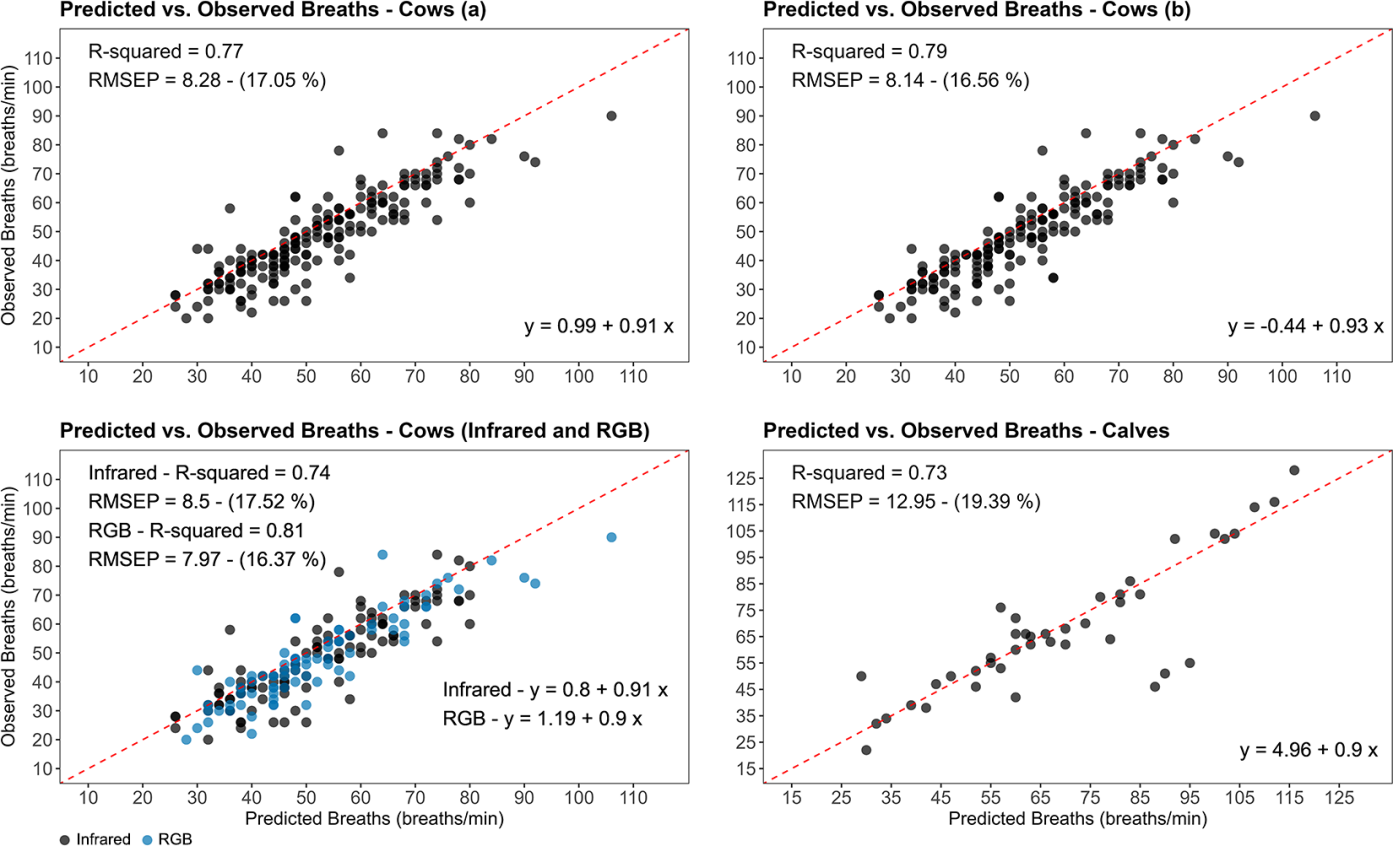
Regression analysis of predicted versus observed breaths. The graphs in the top row represent (a) the entire dataset, composed of 191 observations, and (b) a reduced dataset of 170 observations, excluding observations with non-respiration-related cow movements (e.g., change of position). The graphs in the second row represent (on the left) the entire dataset, analyzing the performance for infrared night vision (blue) and RGB (black) videos separately, and (on the right) the calves' dataset, with 42 observations. The performance metrics are shown in the left corner of the images, where RMSEP is the root mean squared error of prediction. RGB = red, green, and blue.

Considering the reduced dataset comprised of 170 observations, the performance results, as depicted in Figure 2, revealed only a marginal increase in R^2^ (0.79) and a slight decrease in RMSEP (from 8.3 [17.1%] to 8.1 [16.6%] breaths per minute) compared with the previous analysis. These findings indicate that our method may possess sufficient robustness to be unaffected by the previously specified random movements exhibited by the cows within their stalls. This robustness can be attributed to the utilization of FFT to filter out noisy frequencies originating from random movements and isolate the most prominent signals within the data. However, to fully implement our system, an additional step involving automation to accurately capture the ROI during lying time must be developed. Fortunately, this task should not be challenging considering the existing studies that have proposed computer vision approaches for object detection and tracking of animal bodies (Nasirahmadi et al., 2017; Bresolin et al., 2023).

To further evaluate the robustness of our model, we conducted tests to assess its ability to predict the RR for dairy calves using RGB. These calves were housed in different systems and were significantly smaller in size compared with the cows used in the model's development. Despite the differences in dataset, camera, and video acquisition settings, the method consistently and accurately predicted the RR, yielding excellent results. The obtained R^2^ value was 0.73, and the RMSEP was 12.9 breaths per minute, equivalent to 19.4% of the average predicted RR (Figure 1). All previous findings suggest that utilizing FFT on both RGB and IR images to extract frequencies associated with respiration could serve as an alternative method for automated RR monitoring in cattle. This is particularly noteworthy as current studies in this field, such as the work of Lowe et al. (2019), have primarily focused on thermal imaging of nostrils and wearable sensors (Hughes and Iida, 2018).

Considering the satisfying prediction results presented above and the long-distance RR assessment performed with both top-down (for the calves) and angle views (for the cows), the proposed method offers several advantages over other similar technologies. For example, compared with thermal imaging methods that position cameras near the cows' nostrils (Lowe et al., 2019; Jorquera-Chavez et al., 2019) and are susceptible to environmental temperature variations resulting in noisy images (Gade and Moeslund, 2014), our study offers the advantage of significantly reduced chances of animal interaction and device damage during data collection. Additionally, our method was employed using standard security cameras that can be deployed in unrestrained conditions, which better reflects real-world farm settings.

Concerning previous studies using the flank area as a ROI, Wu et al. (2020, 2023) demonstrated a high prediction accuracy of respiration rate through RGB videos. Nevertheless, their study was conducted in a highly controlled environment and employed a sequence of deep learning algorithms to achieve accurate results. For this matter, our study presents the advantage of utilizing FFT for video processing, which eliminates the need for extensive data collection and annotation while potentially improving generalization across diverse environments and imaging conditions. This advantage is especially noticeable with smaller training sets, where training deep neural networks might be impractical or produce models that cannot generalize to new farms or images from different scenes. In our research, the model trained using dairy cows demonstrated effectiveness with calves in both RGB and IR videos.

While it is important to contextualize our findings with prior literature, we must be cautious when making direct accuracy comparisons due to the difference in datasets used to train the models, as well as the validation methods. The lack of benchmark datasets in animal science studies precludes direct method comparisons. A potential solution is to reimplement other methodologies into a study, allowing direct comparisons using identical datasets, if this aligns with the research objectives. However, the animal science field lacks in providing code documentation, which hinders the replication of analyses from published scientific works for direct contrasts. While fields like computer science regularly create and share public repositories such as ImageNet and COCO datasets (Deng et al., 2009; Lin et al., 2014) for developing and evaluating deep neural networks, similar initiatives are absent for animal scientists. In this scenario, it is not appropriate to compare our results with studies that lacked external validation, sourced videos solely from one farm, or assessed RR using images from different body parts (Lowe et al., 2019; Jorquera-Chavez et al., 2019; Wu et al., 2020, 2023). To counter these challenges, Oliveira et al. (2021) suggested establishing communal databases for model validation in precision livestock farming research. Furthermore, Steibel et al. (2023) introduced a Coordinated Innovation Network initiative, aiming to produce expansive, well-annotated image datasets, providing the scientific community with public resources for algorithm development and assessment.

Given that Gaughan et al. (2000) have demonstrated the RR as a reliable indicator of cows' thermal load, the proposed system holds significant potential for monitoring the health conditions of cattle. Ouellet et al. (2021) reported a substantial increase in the RR of dry cows in subtropical weather when the temperature-humidity index exceeds 77, indicating a state of heat stress. However, the traditional visual observation–based method for calculating the RR does not allow for continuous monitoring of the animals' respiratory conditions, underscoring the value of the proposed automated technology as an alternative that enables both accurate RR calculation and consistent monitoring of cows' health status.

Furthermore, evaluating RR may aid in monitoring bovine respiratory disease (**BRD**). Maier et al. (2019) emphasized the importance of a noninvasive system that employs visual scores to diagnose the disease without physically handling the calves. They assessed ambient temperature and clinical signs, including abnormal respiration with increased RR and effort, as predictors of BRD. Animals displaying abnormalities during the initial screening underwent rectal temperature measurement. The model with clinical and environmental signs achieved a sensitivity of 84.2% but a low specificity of 45.7%. However, when rectal temperature was included, the specificity increased to 62.6%. Despite being an advancement in respiratory disease screening, this approach necessitates skilled labor and can be laborious in large herds. Consequently, utilizing the proposed method for continuous RR tracking of resting cattle, in conjunction with other computer vision techniques for behavior tracking, may facilitate the early identification of BRD in these animals.

For future applications, annotating the ROI should not be a significant issue. The model trained to identify the ROI exhibited a precision of 100%, a recall of 71.8%, and an *F*_1_ score of 83.6% for bounding box detection. These results are promising due to high ROI precision. In all predicted images, the bounding box targeted the cow's flank. Despite the satisfying results, we acknowledge that the proposed model must still be further developed for implementation in a commercial setting. Among possible improvements, we highlight the potential of combining the ROI object detector with other detection networks, such as behavior detectors for lying and standing positions. This integration could automate the entire pipeline, enabling decision-making related to changes in respiratory frequency that may contribute to improving animal welfare.

In conclusion, the proposed method demonstrated its robustness by consistently achieving accurate predictive performance across different image types and in unrestrained lying conditions. This approach presents several advantages over other technologies with similar objectives, such as its applicability in unrestrained environments and its ability to generalize well for both cows and calves. Nonetheless, future studies could be conducted to implement disease-detecting algorithms using the proposed method and combine the ROI object detector with other detection networks, thereby facilitating the application of this method in real-world scenarios.
